# Development of an NO_2_ Gas Sensor Based on Laser-Induced Graphene Operating at Room Temperature

**DOI:** 10.3390/s24103217

**Published:** 2024-05-18

**Authors:** Gizem Soydan, Ali Fuat Ergenc, Ahmet T. Alpas, Nuri Solak

**Affiliations:** 1 Department of Metallurgical and Materials Engineering, Istanbul Technical University, Istanbul 34469, Turkey; soydan@itu.edu.tr; 2 Department of Control and Automation Engineering, Istanbul Technical University, Istanbul 34469, Turkey; ergenca@itu.edu.tr; 3 Department of Mechanical, Automotive and Materials Engineering, University of Windsor, Windsor, ON N9B 3P4, Canada; aalpas@uwindsor.ca

**Keywords:** laser-induced graphene, SnO_2_, NO_2_ gas sensor, room temperature gas sensing, environmental monitoring, laser scribing

## Abstract

A novel, in situ, low-cost and facile method has been developed to fabricate flexible NO_2_ sensors capable of operating at ambient temperature, addressing the urgent need for monitoring this toxic gas. This technique involves the synthesis of highly porous structures, as well as the specific development of laser-induced graphene (LIG) and its heterostructures with SnO_2_, all through laser scribing. The morphology, phases, and compositions of the sensors were analyzed using scanning electron microscopy, X-ray diffraction, X-ray photoelectron spectroscopy and Raman spectroscopy. The effects of SnO_2_ addition on structural and sensor properties were investigated. Gas-sensing measurements were conducted at room temperature with NO_2_ concentrations ranging from 50 to 10 ppm. LIG and LIG/SnO_2_ sensors exhibited distinct trends in response to NO_2_, and the gas-sensing mechanism was elucidated. Overall, this study demonstrates the feasibility of utilizing LIG and LIG/SnO_2_ heterostructures in gas-sensing applications at ambient temperatures, underscoring their broad potential across diverse fields.

## 1. Introduction

NO_2_ is one of the most harmful gases to human health and the environment, primarily emitted from the combustion of fossil fuels, biomass burning caused by the heat of lightning during thunderstorms, nitrogen fixation by microorganisms resulting from agricultural fertilization, exhaust from vehicle engines, and emissions from industrial plants [1,2,3]. In addition to causing many environmental issues, such as photochemical smog and acid rain, it can also pose a serious health risk, potentially leading to respiratory conditions such as nose and throat discomfort, bronchitis and pulmonary edema; and in severe cases, it can be fatal [4,5]. The World Health Organization (WHO) emphasizes the importance of monitoring and limiting the concentration of NO_2_ in ambient air, as highlighted in their guidelines for NO_2_ exposure [6]. According to the Occupational Safety and Health Administration (OSHA), the permissible exposure limit (PEL) is 5 ppm for an 8 h time-weighted average (TWA) [7]. The necessity to monitor and detect harmful NO_2_ has prompted improvements in gas sensor technology. These gas sensors play a crucial role in monitoring NO_2_ levels, providing essential data for air quality assessments and ensuring regulatory compliance [6,8].

In gas-sensing technologies, a variety of sensors are employed, with the chemoresistive sensors standing out because of their cost effectiveness, ease of fabrication, and reusability [9,10]. In the field of chemoresistive sensors, semiconductor metal oxides such as TiO_2_ [11], SnO_2_ [12], ZnO [13], WO_3_ [14], Co_3_O_4_ [15], and NiO [16] are notable for their diverse morphologies, thermal stabilities, remarkable surface properties and tunable structures, making them increasingly prominent [2]. Sberveglieri et al. fabricated gas sensors using the single-crystalline SnO_2_ nanobelts with a rutile structure on alumina substrates, capable of detecting a wide range of gases including CO, NO_2_ and C_2_H_5_OH at 200–400 °C [17]. Epifani et al. synthesized SnO_2_ nanocrystals by the sol–gel process (injecting metal oxide sol into a solution of dodecylamine in tetradecane) and deposited them on alumina plates with Pt and Ti/Pt heaters by lithographic techniques to detect NO_2_ gas at 100–300 °C [18]. In addition to these metal oxide-based gas sensors, heterojunctions formed between two metal oxides, such as ZnO-SnO_2_ [19], Sn_3_O_4_-SnO_2_ [20], SnO_2_-In_2_O_3_ have been explored to detect NO_2_ gas [21]. Nevertheless, metal oxide gas sensors typically are not able to work at room temperature because higher temperatures enhance surface reactions such as adsorption and desorption of gas molecules [2,22]. These elevated temperatures also improve selectivity and sensitivity by increasing the mobility of charge carriers. However, the requirement for elevated temperatures necessitates an extra heating element, which increases the operational and maintenance expenditures [23,24].

These challenges have rendered carbon-based gas sensors and their nanocomposites increasingly appealing alternatives in recent years. Carbon materials, such as carbon nanotubes, graphene, graphene oxide, reduced graphene oxide, and their derivatives have demonstrated excellent sensitivity and selectivity in gas-sensing applications. These properties are attributed to their inherent physical and electrical characteristics, including large surface-to-volume ratios, outstanding electrical and thermal conductivities, chemical inertness and high tensile strength—attributes that are particularly pronounced in graphene-based materials [5,23,25,26]. By combining metal-oxide semiconductors with graphene or its derivatives, it is possible to reduce the operational temperature and enhance the sensing performance beyond the capabilities of the individual components. Although intricate and challenging synthesis methods such as two-step laser writing, hydrothermal processes, sol–gel techniques, and electroplating have been used to create graphene-based metal oxide heterostructures, these methods often face challenges in scaling to mass production due to their costly, high-tech requirements and the generation of toxic byproducts [27,28,29,30].

Among these materials, laser-induced graphene (LIG) stands out for its gas-sensing potential, attributed to its unique structure and tunable characteristics, low operational costs, and ease of fabrication [25,31].

This study introduces NO_2_ gas sensors based on LIG and its heterostructure with SnO_2_, designed to function at room temperature. The highly porous structures are synthesized using a one-step laser-scribing process. The operational principles of LIG and LIG/SnO_2_ sensors, based on the adsorption of NO_2_ gas molecules, are elucidated, offering insights into their potential for practical applications in environmental monitoring and public health protection.

## 2. Materials and Methods

### 2.1. Materials

For the fabrication of LIG and LIG/SnO_2_, a ~120 µm thick polyimide (PI) film, sourced from Dupont, was utilized. Tin (II) chloride dihydrate and citric acid monohydrate, both of analytical grade, were procured from Merck for the synthesis.

### 2.2. Chemical and Microscopical Characterization

The morphology and composition of the samples were analyzed using scanning electron microscopy (Axia ChemiSEM, Thermo Fisher Scientific, Waltham, MA, USA). The phase analysis of the samples was conducted by X-ray diffractometer (XRD, Panalytical Aeris, Malvern, UK) with Co-K_α_ radiation. Raman spectroscopy (Renishaw inVia confocal Raman microscope, Dundee, IL, USA) with an excitation wavelength of 532 nm was employed to identify the properties of LIG and its composites. X-ray photoelectron spectroscopy (Thermo Fisher Scientific, K-Alpha X-Ray Photoelectron Spectrometer) was conducted by Al Kα X-ray sources to indicate the surface chemistry and compositions of the samples.

### 2.3. Synthesis of LIG and LIG/SnO_2_

**Figure 1:**
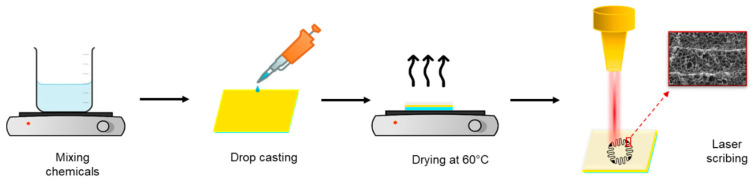
Fabrication of sensor samples.

The synthesis process began with the preparation of a solution for the LIG/SnO_2_ samples by dissolving an appropriate amount of SnCl_2_·2H_2_O in an equal volume of deionized (DI) water. After mixing, citric acid was added to the solution and heated to 80 °C. Subsequently, 100 µL of the solution was drop-cast onto PI film (26 mm × 26 mm), which was bonded to a glass slide and dried on a hot plate at 60 °C. The prepared films were engraved using a commercial laser engraving and a cutting system equipped with 10.6 µm wavelength CO_2_ laser (*P*_max_ = 80 W), operated in raster mode. Then, LIG/SnO_2_ samples were washed with DI water and dried at 100 °C in a drying oven. The flowchart of the fabrication process is given in Figure 1.

Previous studies have demonstrated that PI film, which contains aromatic and imide repeat units, is well suited for forming LIG structures. When the surface of the PI film is irradiated by a laser beam, it can produce high localized temperatures (up to 2500 °C). This photothermal effect, effectively breaks the bonds in PI (such as C-O, C=O, and N-C bonds), allowing the atoms to rearrange. The conversion of sp^3^ carbon bonds into sp^2^ carbon bonds leads to graphitization, particularly in polymers with repetitive aromatic and imide units [32].

The laser parameters were optimized for producing an LIG structure suitable for sensor applications by adjusting the laser power and the degree of defocusing. The specific laser parameters used in this study are presented in Table 1.

First, samples were produced using varying laser powers to fabricate highly graphitized laser-induced graphene with a large specific surface area while maintaining a constant laser-scanning speed, level of defocusing, and pixel density.

**Figure 2:**
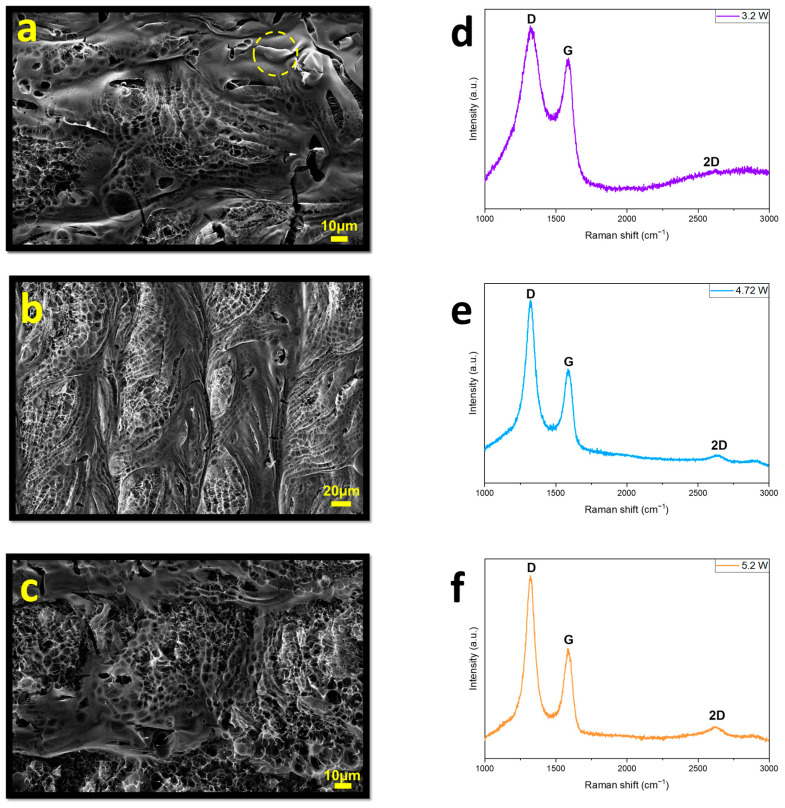
SEM images and Raman spectra of (**a**,**d**) T1, (**b**,**e**) T2, (**c**,**f**) T3.

Raman spectra and SEM images of the samples with increasing laser powers are presented in Figure 2. The morphology of the samples did not exhibit a highly porous structure and certain areas, highlighted with a yellow circle in Figure 2a, did not appear to be fully graphitized. The Raman spectra of the samples exhibited three characteristic peaks located at ~1345, ~1580 and ~2685 cm^−1^, corresponding to the D, G, and 2D peaks. It was observed that the intensity of the 2D peak increased, indicating enhanced graphitization with increasing laser power [33]. Therefore, the laser power was gradually increased to further enhance the intensity of the 2D peak.

However, increasing the power without altering other parameters proved ineffective, as it resulted in delamination.

To achieve a porous structure and more intense 2D peaks, defocusing was optimized. The surface of the PI film reached high localized temperatures exceeding 2500 °C due to laser irradiation. Such intense thermal conditions facilitated the carbonization of the PI film, leading to the emergence of a foamy and porous structure. Moreover, increasing the distance along the z-axis from the focal plane affected the spot size of the laser, causing greater overlap and resulting in the substrate material receiving multiple exposures [32]. These multiple exposures contributed to the formation of a more porous structure. By adjusting the focus to 1 mm above the surface, the spot size was set to approximately 70–80 microns. Subsequently, to fabricate samples with a larger surface area, all laser parameters including laser power, scanning speed, defocusing, and pixel density were optimized as 6.88 W, 40 mm/s, 1 mm (above the surface), and 1000 DPI, respectively. These specific values were configured for the fabrication of gas sensor samples.

### 2.4. Electrical Characterization of the Gas Sensor

**Figure 3:**
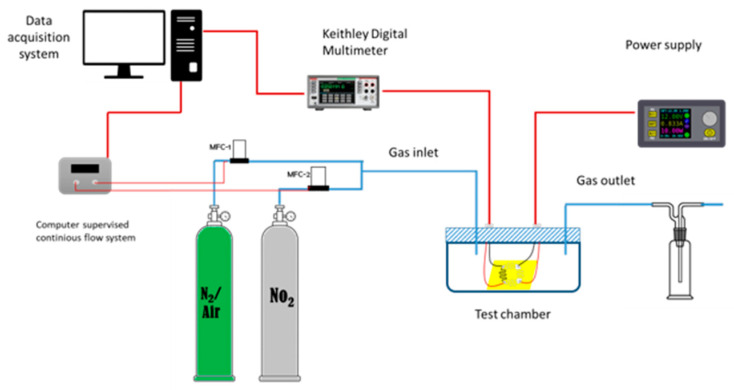
The gas sensor measuring setup.

The electrical characterization of the gas sensor was performed using a custom-made setup, as shown in Figure 3. The sensor was housed inside the gas-tight test chamber equipped with a gas inlet connected to the two mass flow controllers and an outlet connected to a bubbler. The mass flow controllers are operated utilizing an industrial controller (Rockwell PAC ControlLogix, Milwaukee, WI, USA). A gas-tight connector is attached to the top of the chamber to facilitate electrical connections.

**Figure 4:**
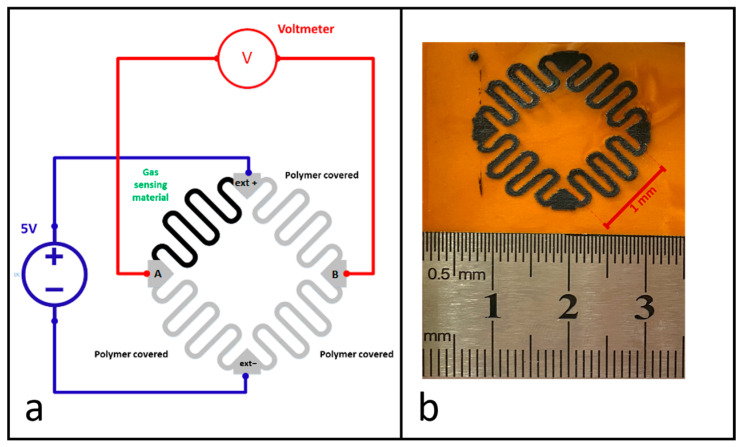
(**a**) The sensor geometry and (**b**) the sensor sample.

The sensor features a rosette-shaped geometry designed to create a Wheatstone bridge to minimize thermal effects, as shown in Figure 4a. An image of the sensor sample is shown in Figure 4b. The Wheatstone bridge sensor configuration includes four identical resistive elements; three are coated with a non-gas permeable polymeric material, and one serves as the gas-sensitive material. The corners of the rosette are covered with silver paste to enable electrical connections. The bridge is powered by a constant 5V bias voltage using an ultra-precise power supply (Keithley 2400 SourceMeter, Cleveland, OH, USA). The balance voltage (V_AB_) of the bridge is measured with a high-precision multimeter (Keithley DMM6500 Multimeter, Cleveland, OH, USA).).

The operation principle relies on the detection of changes in voltage between the measurement points of the bridge due to the resistance variation in the gas-sensitive arm when exposed to the analyte gas. In the characterization experiments, a constant gas flow rate of 1000 standard cubic centimeters per minute (sccm) was maintained. The measurements were conducted under two different atmospheres: N_2_ and air. Prior to conducting the experiments, the sensor sample underwent a 20 min exposure to a flow of N_2_ or air for stabilization. A mixture of the analyte gas and N_2_ or air was then prepared using two mass flow controllers at various concentrations. This mixture flowed over the sample for a duration for 15 min post-stabilization. After each exposure to the analyte gas, N_2_ or air was introduced to flush the chamber for 20 min.

The gas sensor measurements were performed with various concentrations of the analyte gas introduced: 10, 20, 30, 40 and 50 parts per million (ppm). The voltage changes in the sensors were measured in real time and recorded at a rate of 120 data points per minute utilizing a multimeter connected to a computer running Node-Red, Grafana and InfluxDB. The experiment was automated by a program running on Node-Red, a low-code programming environment, which also interfaced with ControlLogix 20.54 for mass flow controller references. The program also managed communication with the multimeter to fetch the measurements. Both flow rates and voltage measurements were collected simultaneously and stored in the InfluxDB database. The data were then visualized using Grafana 10.3.3 software.

The response of the sensor (S) is defined as Δ*V*(*C*) *=* (*V_g_* − *V_i_*)*/V_i_* where *V_i_* represents the voltage without analyte gas exposure and *V_g_* represents the voltage with analyte gas exposure. The change in voltage (Δ*V*) as a function of concentration (*C*) is expressed as a relative change in voltage to minimize the variation between samples. The response time is determined as the time required for the sensor to reach 90% of the total response [34].

## 3. Results and Discussion

### 3.1. Structural and Morphological Characteristics

**Figure 5:**
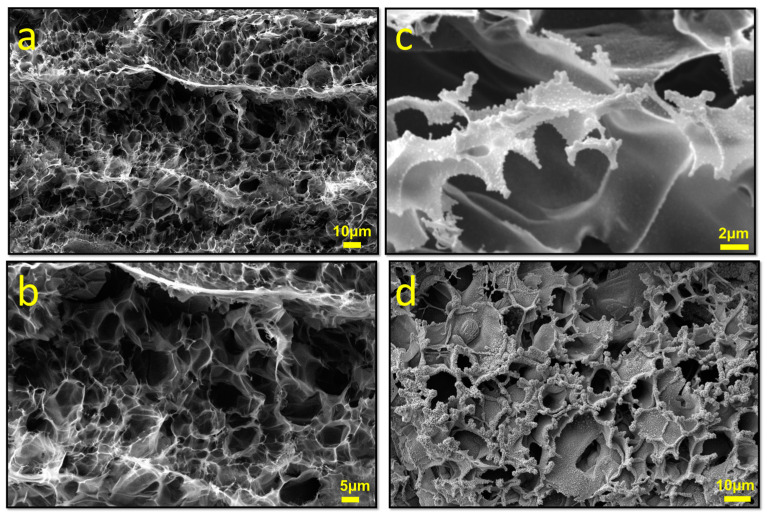
SEM images of (**a**,**b**) the neat LIG and (**c**,**d**) LIG/SnO_2_.

Figure 5a,b shows the highly porous structure of the laser-irradiated neat PI film. The localized high temperatures converted the chloride form of metal on the PI film into its oxide and simultaneously formed a laser-induced graphene structure. This transformation occurred while the surfaces, coated with a gel-like complex solution of metal salt and citric acid, were subjected to laser irradiation. In preliminary attempts to form this heterostructure, SnCl_2_ was dissolved in DI water and applied to the surface of the PI film without the addition of citric acid. However, during the drying process, Sn quickly converted into a metallic salt form and peeled off the surface. As a consequence, a continuous and homogeneous film could not be obtained, and metallic salt remained in the structure shown in Figure S1. The introduction of citric acid transformed the solution into a homogeneous gel, facilitating its application to the PI film’s surface. During drying, no peeling was observed, and a continuous film was formed on the surface. Additionally, upon laser irradiation of the surface, the citric acid promoted the continuation of the combustion reaction, as in the case of sol–gel applications [35].

The in situ fabrication of the LIG/SnO_2_ structure was confirmed by examining the SEM images displaying the morphologies shown in Figure 5c,d; and, with the aid of the EDS spectra and EDS results given in Figure S3a–c and Table S1, the oxide form of Sn was detected in a 3D LIG structure wrapped with SnO_2_ nanoparticles.

**Figure 6:**
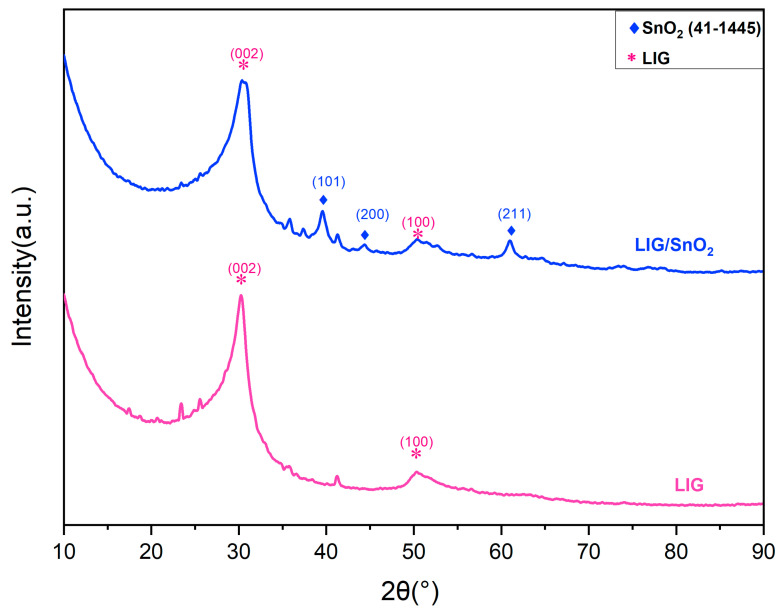
XRD patterns of the neat LIG and LIG/SnO_2_.

XRD patterns of LIG and LIG/SnO_2_ samples are given in Figure 6. Lin et al. suggested that LIG displayed characteristic XRD peaks at 2θ = 25.9° and 42.9°, indicating a high degree of graphitization when analyzed with Cu K_α_ radiation [32]. These peaks corresponded to 2θ = 30.18° and 50.28° for Co K_α_ radiation, which are consistent with the LIG samples. Upon the addition of the Sn complex to the structure, the XRD pattern exhibited additional peaks, in good agreement with cassiterite, SnO_2_ (JCPDS no. 00-041-1445) peaks. This provided additional evidence that laser irradiation not only formed an LIG structure but also created an in situ SnO_2_/LIG heterostructure.

**Figure 7:**
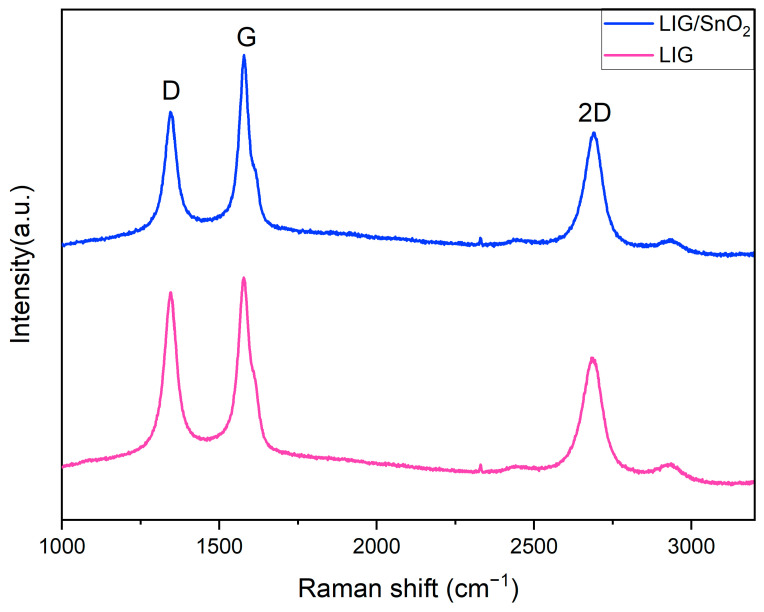
Raman spectra of the neat LIG and LIG/SnO_2_.

In Figure 7, Raman spectra of samples displayed three characteristic peaks of graphene located at ~1345, ~1580 and ~2685 cm^−1^ corresponding to the D, G, and 2D peaks, as mentioned Section 2.2. The D peak indicates imperfections in sp^2^ carbon bonds, the G peak is associated with first-order zone–boundary phonons and the 2D peak arises from second-order zone–boundary phonons. The 2D peaks of samples exhibit only one Lorentzian peak centered at 2700 akin to single-layer graphene with a broader full-width-half-maximum (FWHM). As mentioned before, this method enabled the PI film to reach high localized temperatures; the intensity ratio of the 2D/G peaks indicates a multi-layer graphene structure [33].

According to the literature, the intensity ratios of the I_G_/I_D_ peaks serve as a useful tool for calculating crystallite size (L_a_), with a higher I_G_/I_D_ ratio indicating a larger crystallite size [36]. This is attributed to a higher degree of graphitization resulting from higher surface temperatures. Although all samples were fabricated using the same laser parameters, the I_G_/I_D_ ratio of the neat LIG sample was higher than that of the LIG/SnO_2_. This result suggests that, in the neat LIG sample, the entire laser power was utilized for converting PI to an LIG structure, whereas in the heterostructure, the laser power was also allocated for the formation of metal oxide. Consequently, the proportion of sp^2^ carbon bonds was lower in the heterostructures, leading to lower I_G_/I_D_ ratios.

**Figure 8:**
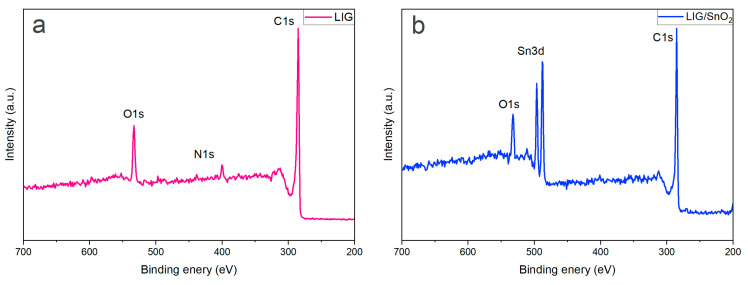
XPS survey spectra of (**a**) the neat LIG and (**b**) LIG/SnO_2_.

The full XPS surveys for both LIG and LIG/SnO_2_ are depicted in Figure 8. For the neat LIG sample, the main peaks belong to C, O, and N. In addition to these, the LIG/SnO_2_ sample exhibits distinct peaks associated with SnO_2_.

**Figure 9:**
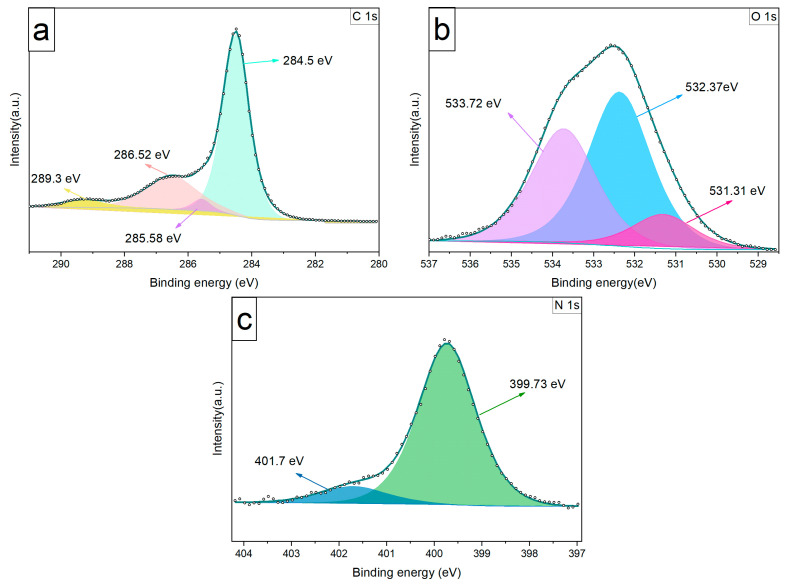
XPS spectra of the neat LIG: (**a**) C 1s spectrum, (**b**) O 1s spectrum, and (**c**) N 1s spectrum.

The C1s spectrum of the neat LIG given in Figure 9 consists of four main peaks. The peaks are located at 284.5 eV, representing the C=C bond, indicating the presence of sp^2^-bonded carbon atoms. Peaks at 285.58 eV and 286.52 eV correspond to C-O-C and O=C-N functionalities, respectively [37]. A peak at 289.3 eV was assigned to O-C=O groups. The prominence of the C=C peak relative to the others suggests that the LIG structure was predominantly composed of sp^2^ carbons in agreement with the Raman results.

From the N 1s spectrum, two nitrogen configurations are identified, namely a peak at 399.73 eV, indicative of pyrrolic nitrogen, and another at 401.7 eV, suggestive of nitrogen in a graphitic environment [37].The O 1s spectrum reveals three oxygen-related peaks. These occur at 531.31 eV for O-C=O, 532.37 eV for C-O-C, and 533.72 eV for C-OH, indicating the presence of various oxygen-containing groups within the material structure [37].

**Figure 10:**
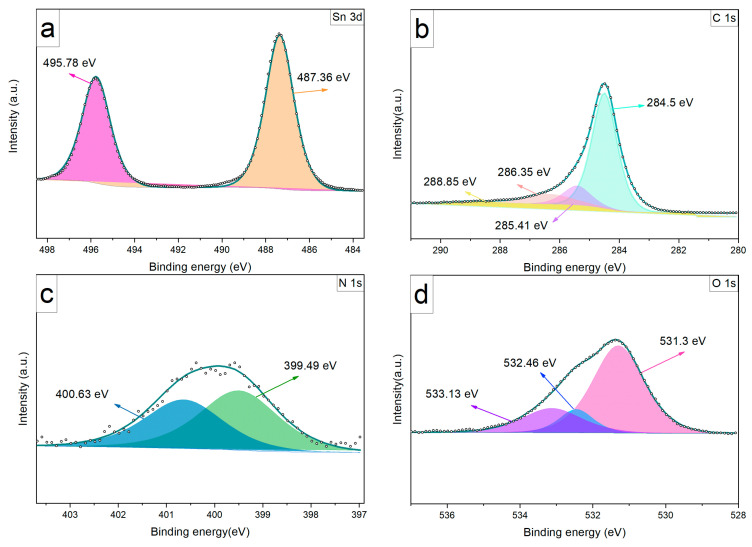
XPS spectra of the LIG/SnO_2_: (**a**) Sn 3d spectrum, (**b**) C 1s spectrum, (**c**) N 1s spectrum and (**d**) O 1s spectrum.

The Sn3d spectrum given in Figure 10 exhibits two characteristic peaks corresponding to Sn 3d_3/2_ and Sn 3d_5/2_ orbitals at 487.36 and 495.78 eV [34]. These results confirm the change in the oxidation state of Sn^2+^ to Sn^4+^ during the laser irradiation.

The C 1s spectrum is composed of four peaks at 284.5 eV C=C, 286.35 eV O=C-N, 285.41 eV C-O-C, and 288.85 eV O-C=O. The C=C peak, having the highest ratio among the peaks, indicates that the LIG structure primarily consists of sp^2^ carbons.

In the N 1s spectrum, two main peaks are located at 400.63 eV and 399.49 eV, corresponding to graphitized and pyrrolic structures, respectively. The O 1s spectrum contains three peaks located at 531.3 eV, 532.46 eV, and 533.13 eV [37]. Liu et al. stated that the O 1s spectrum typically comprises three main peaks from low to high binding energies: crystal lattice oxygen (O_c_), deficient oxygen (O_v_) and adsorbed oxygen (O_ads_) species or OH groups. The O_c_ peak is identified at 533.13 eV, the Sn-O bond at 533.13 eV, while the peaks at 532.46 eV and 533.13 eV are assigned to O_v_ and Oa_ds_, respectively [38,39]. The higher ratio of deficient oxygen (O_v_) in LIG compared to LIG/SnO_2_ suggests that there are more vacancies or defects in the LIG, which can contribute to its p-type semiconducting behavior. The incorporation of SnO_2_, which is an n-type semiconductor, would thus change the overall defect structure and electronic properties of the heterostructure.

Additionally, the results of the N 1s peaks can be interpreted within this context; the ratio of graphitized structures is lower in the LIG than in the SnO_2_/LIG heterostructure, which is indicative of the p-type behavior of graphene structures. It has been suggested that LIG exhibits p-type semiconductor behavior, whereas LIG/SnO_2_ may demonstrate n-type behavior. Furthermore, a slight increase in the binding energies for the Sn 3d orbitals was observed. This upward shift could be attributed to the electron transfer between the metal oxide and LIG structures. This result aids in understanding the heterostructure formed between metal oxide and LIG.

### 3.2. Gas-Sensing Properties

**Figure 11:**
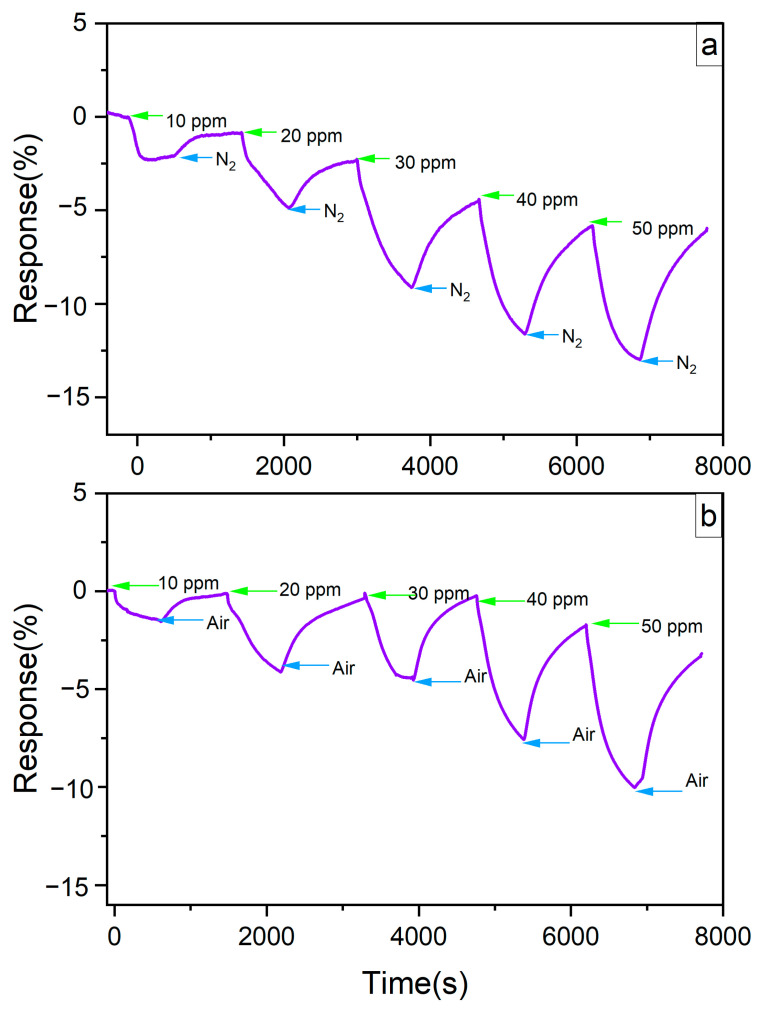
Response time plot of the neat LIG at room temperature in (**a**) inert atmosphere and (**b**) air.

The response time plots of the neat LIG sample at room temperature are presented in Figure 11, showing results when purged with N_2_ (Figure 11a) and with air (Figure 11b). These figures illustrate that, upon exposure to NO_2_ gas, the sensor’s resistance decreased. The response time, defined as the time required for the sensor to reach 90% of the total response, was 435 s for air and N_2_ atmospheres.

**Figure 12:**
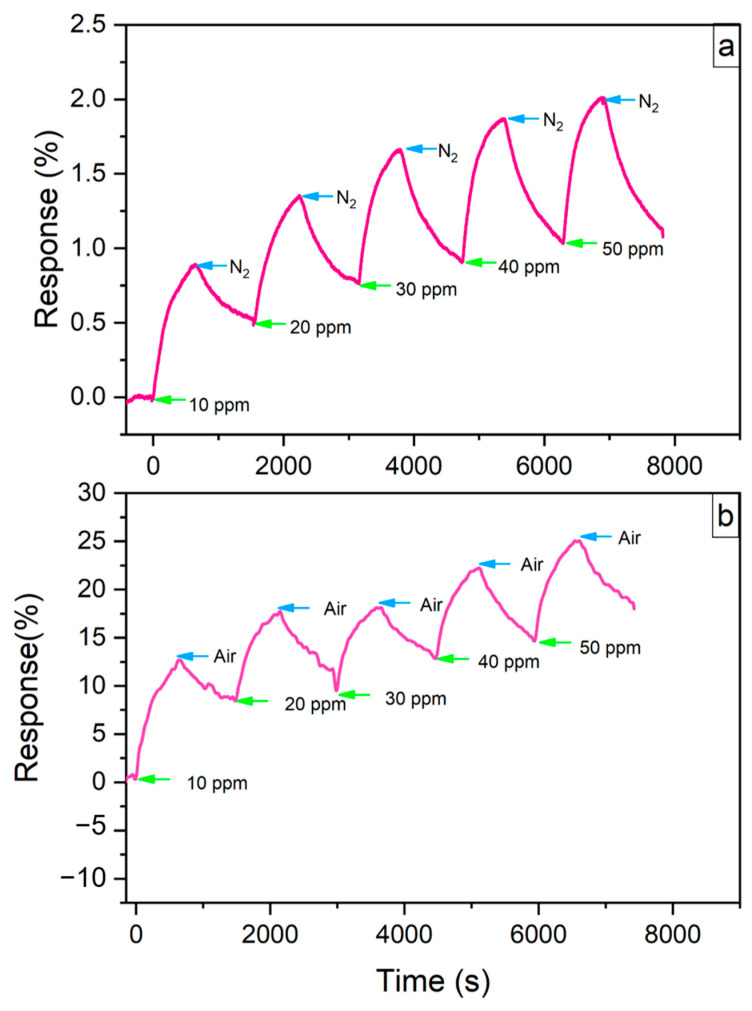
Response time plot of LIG/SnO_2_ at room temperature in (**a**) inert atmosphere and (**b**) air.

Figure 12 presents the response plot for the LIG/SnO_2_ sensor, showing a response time of 475 s for both conditions. Contrary to the neat LIG sensor, the resistance of the LIG/SnO_2_ sensor increased upon exposure to NO_2_. Despite this difference, the LIG/SnO_2_ sensor demonstrated a similar response to both high concentrations and low gas concentrations. This suggests that the LIG/SnO_2_ sensor can detect NO_2_ gas at lower concentrations with greater sensitivity. However, at a gas concentration reaching 30 ppm, the gas sensor sample may become saturated, with all active sites potentially being fully occupied by NO_2_.

The recovery rate of gas sensors was relatively low, with full recovery not observed. This may be due to some NO_2_ gas being trapped within the pores of the LIG, which hinders its immediate release from the sample, a consequence of the material’s porous nature.

**Figure 13:**
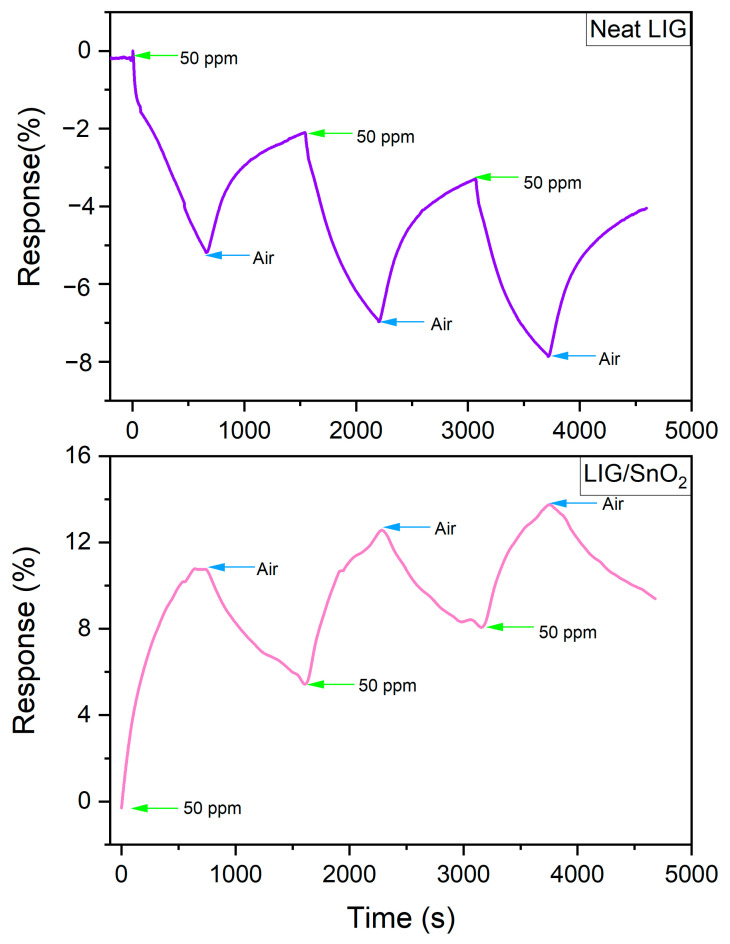
Consecutive measurements of the neat LIG and LIG/SnO_2_ samples toward 50 ppm NO_2_ at room temperature.

To assess the signal reproducibility of sensor materials, consecutive measurements were conducted with 50 ppm NO_2_ at room temperature. As shown in Figure 13, both samples exhibited repeatable responses, indicating reliable sensor performance.

**Figure 14:**
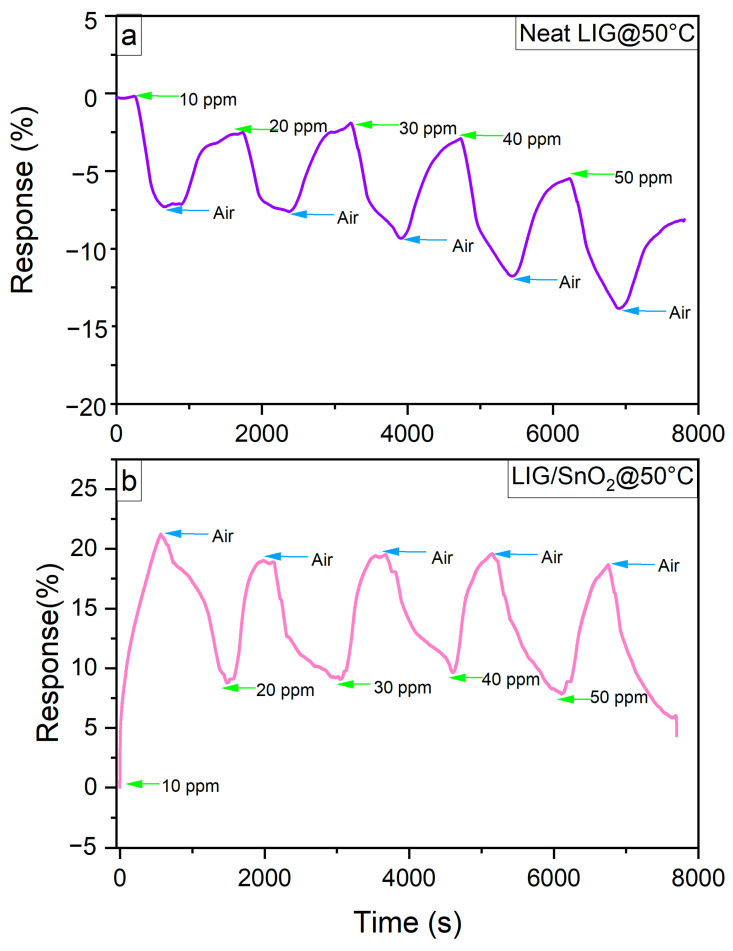
Response time plot of (**a**) Neat LIG and (**b**) LIG/SnO_2_ sample at 50 °C.

To understand the influence of temperature on the recovery rates of the neat LIG and LIG/SnO_2_, samples were tested at 50 °C with NO_2_ concentrations of 10, 20, 30, 40, and 50 ppm. Figure 14a,b presents response time plots of the samples. The neat LIG sample exhibited an increased response compared to its room temperature performance.

Although LIG/SnO_2_ exhibited improved response and recovery rates at this temperature, it did not demonstrate the ability to distinguish between the different concentrations. Similar behavior was observed at room temperature measurements once a certain gas concentration was reached. As previously mentioned, this may be attributed to the equilibrium reached between the adsorption of NO_2_ and O_2_ at the LIG/SnO_2_ interfaces. [40]. While the sensor response is affected by various factors, such as absorbed oxygen species, the rates of adsorption and desorption and the concentration of charge carriers, all of these factors are temperature dependent [41]. As temperature increases, surface reaction rates also increase, causing NO_2_ molecules to compete for adsorption sites. However, as the NO_2_ concentration increases, these adsorption sites may become insufficient, potentially hindering the sensor’s ability to effectively distinguish between different concentrations.

**Figure 15:**
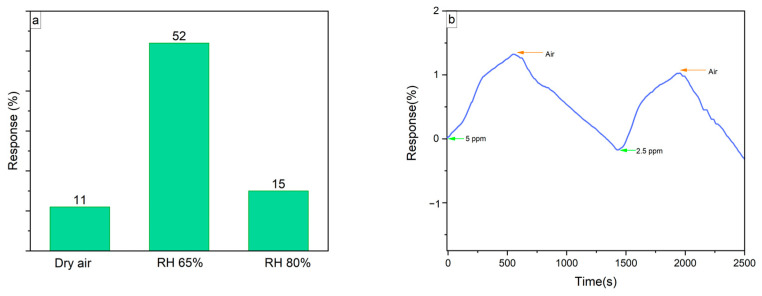
(**a**) The response–RH plot of the LIG/SnO_2_ sample. (**b**) The response-time plot of LIG/SnO_2_ toward 5 and 2.5 ppm NO_2_.

Additionally, the influence of humidity on the samples’ response was examined by measuring the LIG/SnO_2_ samples under various relative humidity (RH) levels in the presence of 50 ppm NO_2_. The results are presented in Figure 15a.

It is apparent that in the presence of high relative humidity, the sensor’s response improved; at 65% RH, the increased water vapor in the air facilitated the adsorption of NO_2_ molecules on the sensor surface, enhancing the sensor response. However, at 80% RH, the response decreased to 15%, which is still better than dry air. This reduction in response at higher humidity levels could be due to the water molecules, leaving fewer sites available for NO_2_ interaction [42]. According to the XPS results, the LIG/SnO_2_ samples included oxygen vacancies and adsorbed oxygen, which promote the absorption water molecules and their decomposition into conductive ions. These ions further decompose more water molecules, thereby increasing the sensor’s sensitivity [42,43].

Furthermore, the LIG/SnO_2_ samples were evaluated for their ability to detect lower concentrations of NO_2_. Successive measurements at 5 ppm and 2.5 ppm are presented in Figure 15b, demonstrating the potential for effective monitoring across a broader range of NO_2_ concentrations.

Selectivity tests with CO_2_ (20,000 ppm) were conducted to evaluate the LIG/SnO_2_ samples’ response. As shown in Figure S3, the LIG/SnO_2_ sensor did not exhibit a considerable response to CO_2_, showing a selective response to NO_2_. Detecting CO_2_ with this type of resistive sensor is more challenging compared to other reducing or oxidizing gases because CO_2_ is chemically inert and less reactive. Therefore, strategies to enhance CO_2_ detection capabilities often involve creating a heterojunction by modifying the surface or synthesizing phase-composited structures, or utilizing higher operating temperatures [44].

### 3.3. Gas-Sensing Mechanism

The gas-sensing mechanism generally consists of three main steps: first, the adsorption of gas molecules onto the sensor surface; second, the transfer of charge from the adsorbed gas; and the third, the desorption of the gas from the sensor surface [20,39]. When exposed to NO_2_ gas, LIG and LIG/SnO_2_ showed two different trends.

In the case of the neat LIG sensor, p-type semiconductor characteristics were evident, with resistance decreasing upon exposure to the oxidizing gas, NO_2_. Laser-induced graphene, synthesized without any oxidizing, reducing, or inert atmosphere generally exhibits p-type semiconductor characteristics, which means that the holes are the primary charge carriers. The adsorption of NO_2_ gas molecules captures these positively charged holes, thus depleting the sensor surface of them. As a result, electron migration from the gas to the sensor surface occurs, leading to a decrease in the sensor’s resistance [34,45].

Conversely, upon exposure to NO_2_, the LIG/SnO_2_ sensor displayed characteristics typical of n-type semiconductors, namely an increase in resistance. This change suggests that the SnO_2_ component significantly influenced the sensor’s electrical behavior, effectively leading to the charge conduction mechanism within the sensor [45]. The enhanced surface area of the heterostructure improved the adsorption of NO_2_ molecules. Typically, n-type semiconductors develop a depletion zone on their surface when exposed to air, as the O_2_ molecules adsorb onto the surface and capture electrons from the materials to form various oxygen species(O_2_^−^,O^−^). This process is shown in Equations (1)–(3) [2].

(1) $$O_{2}\left(gas\right)\to O_{2}\left(ads\right)$$

(2) $$O_{2}\left(ads\right)+e^{-}\to O_{2}^{-}\left(ads\right)$$

(3) $$O_{2}^{-}\left(ads\right)+e^{-}\to 2O^{-}(ads)$$

When the NO_2_ gas is introduced into the chamber, NO_2_ gas interacts with the already formed depletion zone, and it captures additional electrons from the surface. This process is described in Equations (4) and (5).

(4) $${NO}_{2}\left(gas\right)+e^{-}\to NO_{2}^{-}\left(ads\right)$$

(5) $${NO}_{2}\left(gas\right)+O_{2}^{-}\left(ads\right)+2e^{-}\to NO_{2}^{-}\left(ads\right)+2O^{-}(ads)$$

Consequently, the depletion zone widens, leading to a decrease in the concentration of free charge carriers, and ultimately hindering the flow of electricity through the material [46]. Upon the termination of the NO_2_ flow, the material’s surface begins to desorb the gas. Subsequently, the sensor test chamber is purged to remove NO_2_ from the gas chamber.

## 4. Concluding Remarks

This study demonstrates the successful fabrication of flexible gas sensors, capable of operating at room temperature, utilizing LIG and LIG/SnO_2_ heterostructures created through a novel one-step laser-scribing method. Unlike typical metal oxide semiconductor gas sensors that operate at high temperatures, this approach allows for room-temperature functionality, overcoming challenges noted in the existing literature.

The fabrication involved two key strategies. Firstly, it involved using the porous structure of LIG as a template for growing SnO_2_, thereby increasing the surface area through a simple, cost-effective, in situ method. Secondly, a heterojunction was formed between p-type LIG and n-type SnO_2_ to enhance the concentration of charge carriers. In summary, the developed flexible gas sensors based on LIG and LIG/SnO_2_ heterostructures have shown promising results in detecting NO_2_ gas at room temperature. The metal oxide–LIG heterostructure was obtained through one-step laser scribing. The results revealed that LIG/SnO_2_ samples utilized the porous LIG structure effectively as a template. Overall, the LIG/SnO_2_ gas sensor responded to NO_2_ gas well at room temperature. As the temperature increased, the sensor’s response also increased; however, its sensitivity to varying gas concentrations diminished. Moreover, in the presence of humidity, an increase in the response rate was observed. The selectivity test with CO_2_ revealed no significant response, confirming the sensor’s specificity for NO_2_. Given that NO_2_ can serve as a model for oxidizing gas for sensing applications, LIG/SnO_2_ heterostructures hold potential for future applications involving other gases. Furthermore, this one-step laser-scribing method could be adapted to other metal oxides or their complexes for various applications.

## Figures and Tables

**Table 1:** Laser parameters used in the study.

Sample Name	Laser Power (W)	Defocusing (mm)	Scanning Speed (mm/s)	Pixel Density (DPI)
T1	3.2	0	40	1000
T2	4.72	0	40	1000
T3	5.2	0	40	1000
Neat LIG	6.88	+1	40	1000
LIG/SnO_2_	6.88	+1	40	1000

## Data Availability

The data presented in this study are available on request from the corresponding author.

## Supplementary Materials

The following supporting information can be downloaded at: https://www.mdpi.com/article/10.3390/s24103217/s1, Figure S1. LIG/SnO_2_ morphology without the addition of citric acid, Figure S2. (a,b) EDS spectra and (c) SEM image of LIG/SnO_2_, Table S1. EDS results of LIG/SnO_2_, Figure S3. The response-time plot of LIG/SnO_2_ sample toward 20,000 ppm CO_2_.
