# Supplementary material for: Development of an NO2 Gas Sensor Based on Laser-Induced Graphene Operating at Room Temperature
Source: Sensors (Basel). 2024 May 18;24(10):3217. doi: 10.3390/s24103217 (PMC11125758; doi:10.3390/s24103217)
Supplement: Supplementary file 1 [file sensors-24-03217-s001.zip › sensors-2982268-supplementary.pdf]

sensors

Supplementary Materials for

Development of an NO_2 Gas Sensor Based on Laser-Induced Graphene Operating at Room Temperature

Gizem Soydan [1], Ali Fuat Ergenc [2], Ahmet T. Alpas [3] and Nuri Solak [1,*]

[1] Department of Metallurgical and Materials Engineering, Istanbul Technical University, Istanbul 34469, Turkey; soydan@itu.edu.tr
[2] Department of Control and Automation Engineering, Istanbul Technical University, Istanbul 34469, Turkey; ergenca@itu.edu.tr
[3] Department of Mechanical, Automotive and Materials Engineering, University of Windsor, Windsor, ON N9B 3P4, Canada; aalpas@uwindsor.ca
* Correspondence: solaknu@itu.edu.tr

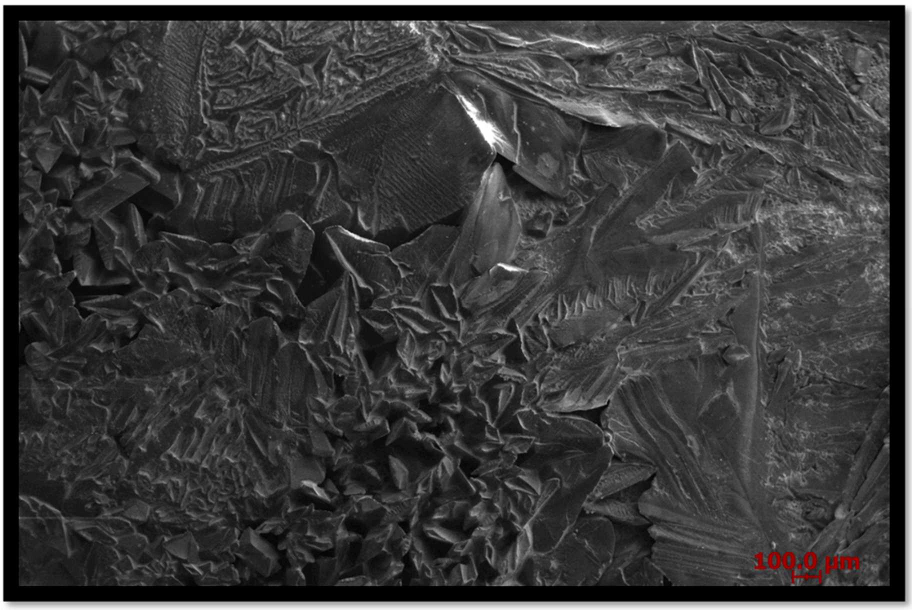

Figure S1. SEM image of SnO_2/LIG structure without citric acid.

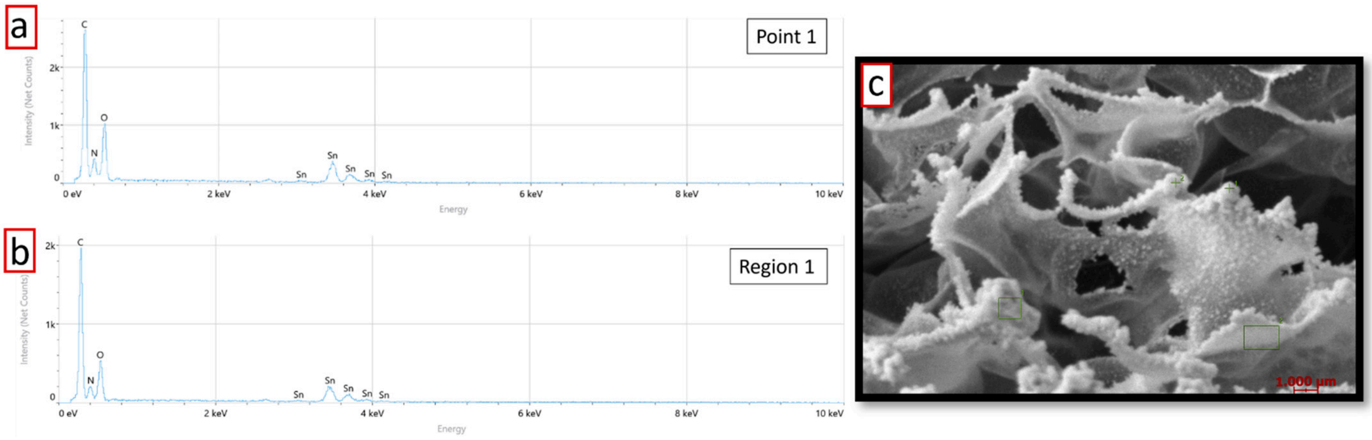

Figure S2. (**a**,**b**) EDS spectra and (**c**) SEM image of LIG SnO_2.

Table S1. EDS results of LIG/SnO_2.

Element	Point 1		Region 1	
	Atomic %	Weight %	Atomic %	Weight %
C	40.5	18.0	44.5	20.0
N	5.1	2.6	5.6	3.0
O	42.1	25.0	37.7	22.6
Sn	12.3	54.3	12.2	54.4

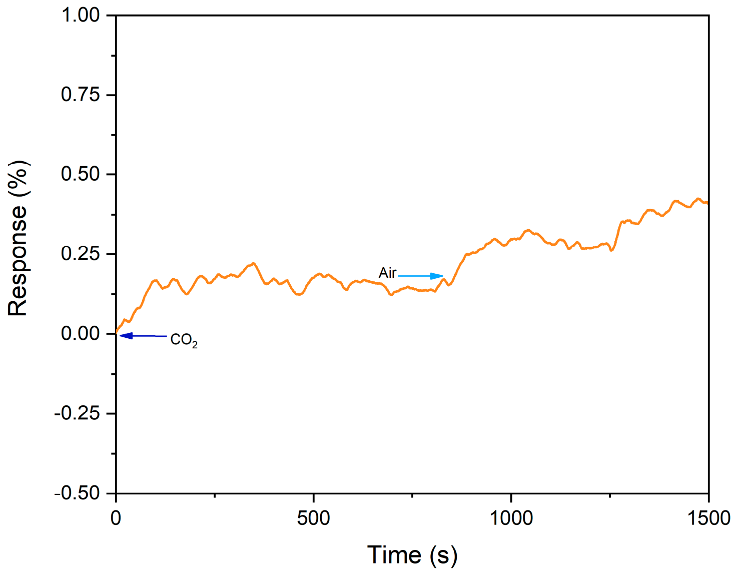

Figure S3. The response-time plot of LIG/SnO_2 sample towards 20,000 ppm CO_2.
